# Systemic Sclerosis Associated Interstitial Lung Disease: New Directions in Disease Management

**DOI:** 10.3389/fmed.2019.00248

**Published:** 2019-10-31

**Authors:** Mehdi Mirsaeidi, Pamela Barletta, Marilyn K. Glassberg

**Affiliations:** Division of Pulmonary, Critical Care, and Sleep Medicine, University of Miami Miller School of Medicine, Miami, FL, United States

**Keywords:** scleroderma, interstitial lung disease, systemic sclerosis, cyclophosphamide, nintedanib, pirfenidone

## Abstract

A subgroup of patients with systemic sclerosis (SSc) develop interstitial lung disease (ILD), characterized by inflammation and progressive scarring of the lungs that can lead to respiratory failure. Although ILD remains the major cause of death in these individuals, there is no consensus statement regarding the classification and characterization of SSc-related ILD (SSc-ILD). Recent clinical trials address the treatment of SSc-ILD and the results may lead to new disease-altering therapies. In this review, we provide an update to the diagnosis, management and treatment of SSc-ILD.

## Introduction

Scleroderma or systemic sclerosis (SSc) is a systemic multi-organ disorder characterized by autoimmunity, systemic inflammation, vascular injury, and tissue fibrosis (1). Hippocrates provided the first description of their “thickened” skin texture around 400 BCE followed by labeling of the skin as “wood-like” by Curzio (2). In 1836, Fantonetti applied the term “scleroderma,” derived from the Greek words skleros (hard or indurated) and derma (skin), to describe the human skin and joint disease presenting with tightened dark leathered skin leading to impaired joint mobility (2).

Classification of patients with SSc is based on the extent of skin involvement- diffuse cutaneous sclerosis (dcSSc) or limited cutaneous sclerosis (lcSSc), the latter characterized by skin sclerosis restricted to the hands, face, neck and distal extremities (3). Although SSc mainly affects the skin, pulmonary manifestations have an unpredictable course and remain the main cause of morbidity and mortality (4).

## Epidemiology

The overall incidence rate of SSc in the adult population of the United States is approximately 20 per million per year (5) and approximately one in 10,000 individuals worldwide (1). Incidence and prevalence rates are fairly similar for Europe, the United States, Australia, and Argentina suggesting a prevalence of 150–300 cases per million; Scandinavia, Japan, the UK, Taiwan, and India report lower prevalence (6). The European League Against Rheumatism (EULAR) study showed a median disease duration of 7.1 years for patients with dcSSc and 15.0 years for lcSSc (7). The ratio of women to men developing SSc is 4:1 with an age of 45–55 at presentation (8). Cigarette smoking contributes to disease severity, but is not associated with risk of developing SSc-ILD (9).

In a review of patients with SSc-ILD, pulmonary fibrosis accounted for 19% of deaths and pulmonary hypertension (PH) in 14% (4). In an Italian cohort, the survival of SSc-ILD patients was reported to be 29–69% at 10 years from diagnosis with a female to male ratio of 9.7:1 (10). African-American scleroderma patients have an earlier onset and more severe pulmonary disease. However, African American race is not a significant risk factor for mortality after adjustment for socioeconomic factors (11). Al-Sheikh reported that European-descent white subjects (55%, 95% CI 51–60) have poorer survival compared to Hispanic subjects (81.3%, 95% CI 63–100). East Asians have the longest median survival time (43.3 years) and Arabs the shortest median survival time (15 years) (12). Independent of race, lower median household income predicted increased mortality (11).

## Mechanism of Fibrosis in SSc-ILD

Similar to other fibrotic lung diseases, injury to epithelial cells, activation of innate and adaptive immunity, and fibroblast recruitment and activation may lead to excessive extracellular matrix production and scarring in SSc-ILD (13). The factors that promote the activation and increased matrix production of fibrogenic fibroblasts in SSc-ILD are not well studied. However, recent data suggest that myofibroblast differentiation and proliferation are key pathological mechanisms driving fibrosis in SSc-ILD (14).

In bronchoalveolar lavage (BAL) fluid from patients with SSc-ILD, the pro- inflammatory cytokines interleukin (IL)-8, tumor necrosis factor-a (TNF), and macrophage inflammatory protein−1a are increased (15). Lung biopsies from patients with SSc-ILD demonstrate increased expression of Toll-like receptor (TLR) 4 in fibroblasts (11, 16). TLR4 is widely recognized as central to the innate response to gram-negative bacteria, but it can also be activated by endogenous ligands generated by cellular injury, autoimmune response, and oxidative stress. TLR4 activation potentiates TGF–β signaling and suppresses antifibrotic microRNAs (miR-101, miR 18a5p, miR-1343, miR-153, miR-326, miR-27b, miR-489, miR26a) (11, 17). TGF-β, through indirect influence on cytokines, primarily platelet derived growth factor (PDGF), promotes fibrogenesis (18). Elevated levels of IL-33 have been correlated with the severity of skin and lung fibrosis (19).

## Other Pulmonary Manifestions in SSc

Lung involvement including ILD, PH, or a combination of ILD and PH, occurs in more than 70% of patients with SSc. Pulmonary vascular disease, primarily pulmonary arterial hypertension, occurs in 10–40% of patients with SSc. Recently, coexisting PH was reported in a large SSc-ILD cohort often occurring early after diagnosis of SSc-ILD (20).

## Clinical Diagnosis of SSc-ILD

The diagnosis of SSc-ILD is based on finding ILD on HRCT of the chest in a patient with known SSc accompanied by normal or abnormal pulmonary function tests showing restriction. Approximately one third of patients with SSc have positive anti-topoisomerase (Scl-70) antibodies; these patients have a greater likelihood of developing ILD, compared to those with lcSSc or those with positive anti-centromere antibodies (21). In the EULAR analysis, 53% of cases with dcSSc and 35% of cases with lcSSc had SSc-ILD (22). Historically, African American ethnicity, higher Rodman skin score (a measure of skin thickness), high creatinine and serum CPK levels, hypothyroidism, and cardiac involvement are associated with increased risk for the development of ILD (23, 24). Current risk factors for progression include diffuse vs. limited disease, a disease duration of >5 years, extent of parenchymal disease on HRCT of >20%, a forced vital capacity (FVC) of <70%, and the detection of anti-topoisomerase antibody (25).

## Diagnosis of SSc-ILD

The most common symptoms of SSc-ILD are dyspnea, fatigue, and non-productive cough (26). Early ILD is frequently asymptomatic. As part of the diagnostic evaluation for a patient with SSc-ILD, auscultation of bibasilar fine inspiratory crackles at the lung bases should warrant a HRCT of the chest (27). The most common radiological finding is a non-specific interstitial pneumonia pattern with peripheral, bibasilar distribution of ground glass opacities (28, 29) (**Figure 2**). A pattern of usual interstitial pneumonia, characterized by honeycomb cysts and traction bronchiectasis may also be seen in up to a third of patients with SSc-ILD (29). The presence of ground glass opacities may herald the development of pulmonary fibrosis (30).

The most common histopathologic finding on lung biopsy is fibrotic NSIP (31) (Figure 3). A usual interstitial pneumonia (UIP) pattern can also be seen. When compared to lung biopsies of patients with idiopathic pulmonary fibrosis, SSc-ILD patients have more germinal centers and fewer fibroblast foci (32).

Almost all patients with SSc-ILD have positive antinuclear antibodies; this can be accompanied by anti-topoisomerase I (anti-Scl-70), anti- Th/To, anti-U3 ribonucleoprotein (RNP), anti- U11/U12 RNP, and rarely anti-centromere antibodies (33). The sensitivity and specificity of these autoantibodies varies in SSc depending on ethnicity, geographic region of origin, and method of detection (34).

Pulmonary function tests may be normal at presentation, but can be helpful in the follow up of SSc-ILD (35). Forced vital capacity below 80%, low diffusing capacity of the lungs for carbon monoxide (DLCO), and older age are predictors for mortality in SSc-ILD (15, 36). A rapid decline in DLCO may be the single most significant predictor of poor outcome and extent of ILD (37–39).

Analysis of BAL from patients with SSc-ILD typically shows increased number of granulocytes, especially neutrophils and eosinophils, and sometimes an increased level of lymphocytes and mast cells (40). In a series of 156 patients with SSc-ILD, a high percentage of neutrophils in BAL was associated with a 30% increase in risk of mortality (41).

The diagnosis of SSc-ILD is based on finding ILD on the HRCT of the chest in a patient with known SSc, and with exclusion of other etiologies of pulmonary parenchymal disease such as drug induced lung toxicity, heart failure, or recurrent aspiration. A lung biopsy may be considered if there is suspicion for malignancy or granulomatous disease (40).

## Biomarkers in the Diagnosis of SSc-ILD

There are no biomarkers that are part of a standard of care diagnostic work-up. In two study cohorts that included 427 individuals with SSc, lung-epithelial-derived surfactant protein (SP-D) was identified as a potential biomarker of SSc-ILD. It is suggested that elevated serum levels of SP-D would increase the risk of finding pulmonary fibrosis on chest images 3-fold (OR: 3.15 [1.81–5.48], *p* < 0.001) (42). Chemokine (C-C motif) ligand 18 (CCL18) is another biomarker that may predict the progression of ILD. The CCL18 is a pro-fibrotic factor and is found elevated in serum, BAL and lung tissue from patients with IPF or SSc-ILD (43). CCL18 is secreted predominantly by alveolar macrophages and is reflective of active lung injury (44).

The levels of Krebs von den Lungen-6 (KL-6), a glycoprotein found predominantly on type II pneumocytes and alveolar macrophages, are elevated in the serum of patients with SSc-ILD and may correlate with the presence of pneumonitis and the radiological fibrosis score in patients with SSc (45). KL-6 has been used as a marker for acuteness of lung fibrosis and the presence of pneumonitis (42). In a study of lung biopsies from 112 patients, the KL-6 level was significantly higher in patients with clinically active pneumonitis (1,497 +/- 560 U/ml) compared with inactive pneumonitis (441 ± 276 U/ml (*p* < 0.001) (46).

## Clinical Management of Patients With SSc-ILD

The importance of a decline in lung function and survival in patients with SSc was noted by Ferri (47). SSc-ILD is classified as limited or extensive based on the findings of high-resolution computed tomography (HRCT) and lung function FVC (15). Patients with >20% HRCT abnormalities are considered to have extensive lung disease and those with <20% HRCT changes as limited disease. If the FVC is <70%, patients have extensive lung disease, and if the FVC is >70%, patients have limited disease (15). Patients with extensive disease have higher mortality and risk of lung function deterioration (15).

The treatment for SSc-ILD has focused on immunosuppressive therapies, particularly cyclophosphamide (CYC) and mycophenylate mofetil (MMF) based on the results of two pivotal clinical trials. Results from the Scleroderma Lung Study 1 showed a 1% change in FVC in the placebo group compared to a 2.6% change in FVC in the treated SSc subjects at 12 and 18 months (31). After 24 months, there were no differences between groups (48, 49). The results of the Scleroderma Lung Study I supported CYC as a standard of care until smaller studies reported beneficial effects of MMF in SSc-ILD. This led to the Scleroderma Lung Study II comparing CYC vs. MMF showing that MMF was as effective and safer than CYC over a 24-month time period (54). Although this trial had a large dropout rate and lacked a placebo arm, MMF fell into a standard of care for SSc-ILD (54). Goldin et al. recently reported that changes in quantitative fibrosis scoring of the HRCT in SLS II correlated with FVC and the transition dyspnea index (50).Despite a previously negative trial with a tyrosine kinase inhibitor, imatinib (51), the recently completed SENSCIS trial in which 50% of the subjects were on a stable dose of MMF demonstrated an improvement in FVC with the addition of nintedanib (52). Of note, 50% had diffuse SSc and 60% of the participants were anti-topoisomerase positive.

**Figure 1:**
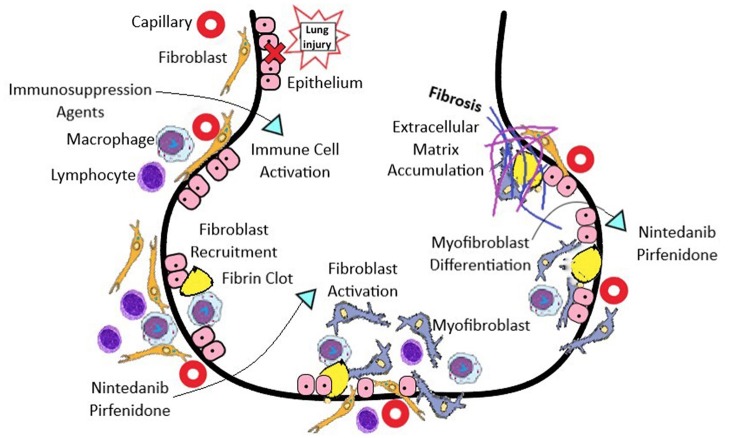
The pathogenesis of SSc-ILD involves vascular, immunological, and fibrotic processes. The initial injury begins with endothelial and alveolar cell injury, which upregulates adhesion molecules and chemokines to attract leukocytes, which enable both innate and adaptive immune responses. Anti-topoisomerase 1 antibodies form immune complexes, and are taken up via Fc receptors, and activate endosomal Toll-like receptors in immune cells, which leads to type I interferon production. IFN release can induce TLR 3 expression on the surface of fibroblasts, causing pro-collagen production. Ligands for Toll-like receptors (TLRs) stimulate dendritic cells to produce IFN-α and interleukin (IL)-6, which in turn activate Th2 cells, produce IL-4 and IL-13, and stimulate pro-fibrotic macrophages. Macrophages produce multiple profibrotic factors including: TGFβ, connective tissue growth factor (CTGF), and PDGF, which promote fibroblast recruitment, invasion and proliferation. Fibroblast activation then occurs, and differentiation to a contractile myofibroblast phenotype result in overproduction and accumulation of extracellular matrix, resulting in progressive fibrosis. ^*^Immunosuppression agents: mycophenolate mofetil, cyclophosphamide, tacrolimus, cyclosporine, tocilizumab, rituximab.

**Figure 2:**
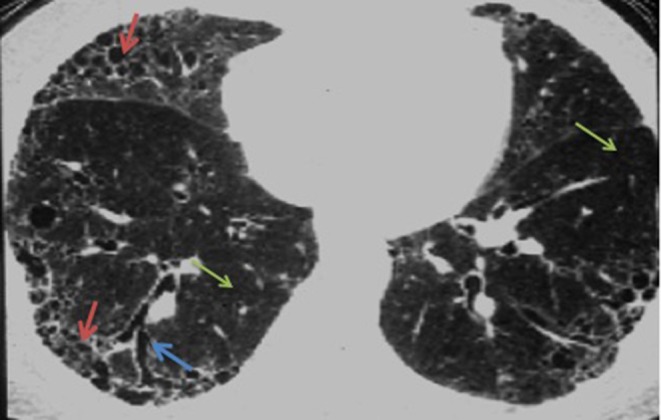
HRCT of a usual interstitial pneumonia (UIP) pattern, characterized by honeycombing (red arrows), and traction bronchiectasis (blue arrow). Normal lung tissue is signaled with green arrows.

The optimal treatment of SSc-ILD is not known. Developing treatments that would prevent SSc-ILD disease progression rather than disease regression is a research goal (39). Current management includes initiation of immunosuppressive treatment for SSc-ILD with ongoing evidence of disease progression based on PFT decline or radiographic deterioration. Initial therapy does not include steroids in light of the risk of renal crisis especially in dsSSc patients. Patients are more likely to benefit from immunosuppressant therapy during the early course of the disease, before substantial loss of lung function occurs (53). The most rapid decline in FVC occurs within the initial 3 years of disease onset (54). When therapy is initiated, exercise tolerance and PFTs should be monitored at 6-month intervals (55). Frequent HRCT images are not recommended and can be repeated when a change in clinical symptoms occur. (56) Most physicians seem to treat patients with extensive lung disease (presentation in HRCT and lung biopsy with UIP pattern, and evidence of ground glass opacities occupying more than 10% of lungs (Figures 1, 2). With the completion of more randomized clinical trials, newer treatments with or without the adopted immunosuppressive agents may demonstrate efficacy in SSc-ILD.

**Figure 3:**
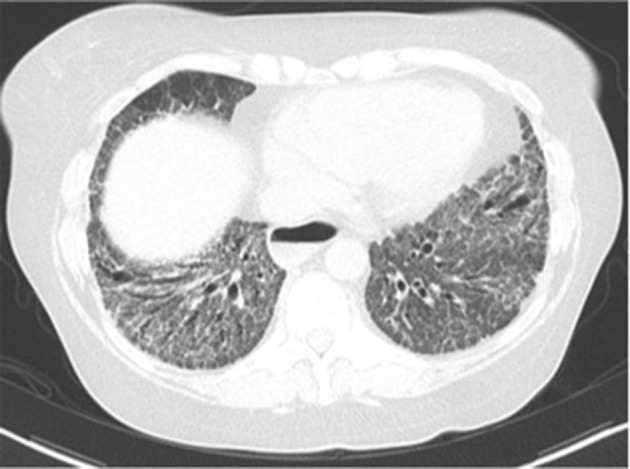
Fibrotic nonspecific interstitial pneumonia (NSIP).

### Mycophenolate Mofetil (MMF)

Mycophenolate mofetil (MMF)is an inhibitor of lymphocyte proliferation and is often used as first line treatment in patients with SSc-ILD who are at risk for progressive ILD (57). The role of MMF in SSc-ILD was studied in the Scleroderma Lung Study II that evaluated 142 patients with SSc-ILD with FVC of <80%, and ground glass opacities on HRCT. Participants were given either 1,500 mg MMF twice daily for 24 months or oral cyclophosphamide (CYC) titrated up to a maximum dose of 1.8–2.3 mg/kg for 12 months. MMF was better tolerated than CYC and had a lower incidence of leukopenia and thrombocytopenia (57). Bone marrow suppression and gastrointestinal (GI) symptoms were the most commonly observed adverse effects of MMF. A complete blood count should be performed before starting therapy and during treatment. The target dose of MMF is generally between 1.5 and 3 g daily usually in two divided doses to avoid GI side effects.

In an observational study, 13 patients received anti-thymocyte globulin plus prednisolone for 5 days, followed by MMF maintenance therapy for 12 months. Long-term MMF was well tolerated, but there was no change in mean FVC or diffusion capacity after receiving this combined therapy (58).

### Cyclophosphamide (CYC)

Cyclophosphamide (CYC) is considered an alternative to MMF based on the results of the Scleroderma Lung Study II. The unfavorable adverse effect profile includes infertility, opportunistic infections, hemorrhagic cystitis, bladder cancer, and neutropenia (59). Monthly intravenous administration of CYC is preferred over oral administration, due to a lower cumulative dose effect, less frequent adverse effects, and the ability to ensure adequate hydration before administration (28). Six CYC monthly intravenous infusions are recommended (60), with monthly monitoring of white blood cell count, renal function, and urinalysis. Corticosteroid pulses have been used with CYC with favorable results, but not as monotherapy (61). After completing a course of CYC, the treatment is commonly switched to a less toxic maintenance agent such as MMF or Azathioprine. Improvement in lung function after CYC treatment tends to decrease after discontinuation (62). For this reason, maintenance therapy is recommended, preferably with MMF (57).

### Azathioprine

Azathioprine is a less efficacious initial therapy for SSc-ILD than CYC. In a randomized, double-blind trial, 60 patients with early SSc-ILD received either Azathioprine or CYC. During the first 6 months of therapy, patients also received prednisone, which was tapered subsequently. After 18 months FVC (−11.1 ± 1%), and DLCO (−11.6 ± 1.3%) were significantly worse (*p* < 0.001) in the Azathioprine group. In the CYC group, DLCO and FVC remained unchanged (63).

### Cyclosporine and Tacrolimus

Cyclosporine and tacrolimus selectively inhibit calcineurin, thereby impairing the transcription of IL-2 and several other cytokines in T lymphocytes. Cyclosporine is an immunosuppressive agent mainly used to treat organ rejection post- transplant. Cyclosporine is a highly nephrotoxic agent that causes a decrease in the glomerular filtration rate (GFR) and a decrease in creatinine clearance (64). In a retrospective, observational study, tacrolimus may have some benefits for SSc-ILD. Twenty patients with SSc-ILD treated with CYC were divided into two groups: one treated with tacrolimus and low-dose corticosteroids following CYC and the other treated with low-dose corticosteroids after CYC. No difference was observed in PFTs at baseline in each group (%VC: 79.5 ± 16.1% vs. 87.4 ± 18.8%, %DLCO: 59.5 ± 11.5% vs. 63.7 ± 14.6%). In 3 years follow up; subjects treated with tacrolimus did not demonstrate disease progression (65). Neither CYC or tacrolimus is considered standard of care management for SSc-ILD.

### Bosentan

Bosentan, is a nonselective endothelin receptor antagonist, used in the treatment of pulmonary hypertension. It is known that the endothelin system participates in the pathogenesis of SSc, and that it could delay the progression of SSc-ILD. A prospective, double-blind, randomized, placebo-controlled, parallel group study was conducted to evaluate changes in 6 min walk test distance, FVC and DLCO changes. 163 patients were enrolled, 77 were randomized to receive Bosentan, and 86 were randomized to receive placebo for 12 months. No significant difference between treatment groups was observed for change in the 6-min walk distance. No deaths occurred in this study group. FVC and DLCO remained stable. In Conclusion, these data do not support the use of endothelin receptor antagonists as therapy for SSc-ILD (66).

## Biological Immunotherapies

### Rituximab

Rituximab, a monoclonal antibody that targets CD20 positive B-lymphocytes, is suggested for patients with refractory SSC-ILD (67). In a pilot study, rituximab plus standard therapy (prednisone, CYC, and/or MMF) compared to standard therapy alone showed that the 8 patients in the rituximab group had a significantly better FVC, and DLCO (median percentage of improvement of 10.25 and 19.46%, respectively) at 1 year, than the other 6 patients receiving standard therapy alone (68). Further studies are need to assess the efficacy of rituximab in SSc-ILD (69).

### TociIizumab

TociIizumab, a humanized monoclonal antibody against the human IL-6 receptor *a* chain, is approved for treatment of rheumatoid arthritis, juvenile idiopathic arthritis, and Castleman's disease (70). In patients with SSc-ILD, higher levels of serum IL-6 appear to be predictive of early disease progression in patients with mild ILD, this could be used to target treatment in this group of patients (71). In a randomized 48-week trial of 87 patients with dcSSc, FVC was significantly improved after 24 weeks in the Tocilizumab group (−34 vs. −171 ml respectively, *p* = 0.0368). However, no significant difference in FVC was found between the treated and control groups at 48 weeks (72).

### Pomalidomide (POM)

Pomalidomide (POM), is an immunomodulator with antiangiogenic properties, and cytotoxic activity. Approved for the treatment of relapsed and refractory multiple myeloma (73). A 52 week randomized, double blind clinical trial of 23 patients with SSc-ILD was conducted to evaluate the safety and efficacy of POM on FVC and mRSS. Twenty-three patients were enrolled and randomized to receive POM or placebo. FVC deteriorated in both treatments (POM −5.2%, placebo −2.7%), mRSS (POM −2.7, placebo −3.7). Since very few subjects were enrolled the results were inconclusive (74).

### Bortezomib

Bortezomib, is a FDA approved medication for the treatment of multiple myeloma. Bortezomib inhibits TGF- signaling *in vitro*, promotes normal repair and prevents lung fibrosis. The objective of the trial is to establish the safety and tolerability of bortezomib in SSc patients as well as exploratory effects on FVC. Participants receive MMF (1.5 g twice a day orally) and Bortezomib(1.3 mg/m^2^) subcutaneously once per week for the first 2 weeks vs. MMF plus placebo (normal saline) for 24 weeks. The trial is planned for completion in June 2019.

## Anti-fibrotic Agents

Nintedanib and pirfenidone have anti-fibrotic effects and are approved for use in patients with idiopathic pulmonary fibrosis (IPF). In a case series of five patients with SSc-ILD, pirfenidone (1,200–1,800 mg/day) was associated with a reduction in dyspnea and an increase in VC (10%) from baseline (75). LOTUSS, a 16-week open label phase II trial of the safety and tolerability of pirfenidone on patients with SSc-ILD, pirfenidone was generally well tolerated, but there were no significant changes in FVC (76). SLS III, a double-blind, parallel group, randomized and placebo-controlled clinical trial is currently being conducted in patients with SSc-ILD. Participants must be treatment naive. The objective of this study is to determine the efficacy and safety of the combination of MMF with Pirfenidone. Subjects will be randomized 1:1 to receive MMF plus Pirfenidone or MMF plus placebo. The trial is scheduled for completion on May 2021.

Nintedanib, is a tyrosine kinase inhibitor (77) for vascular endothelial growth factor (VEGF), fibroblast growth factor (FGF), and platelet-derived growth factor (PDGF), and colony stimulating factor 1 receptor (CSF1R) (78), slows disease progression and improves survival in patients with IPF. The SENCSIS trial, a double blind, randomized, placebo-controlled trial evaluated the efficacy and safety of oral nintenadib (150 mg bid) treatment for at least 52 weeks in patients with SSc-ILD (79). In the SENSCIS trial, 50% of the subjects had dsSSc and were on a stable dose of MMF. Subjects had a diagnosis of SSc with an onset of the first non-Raynaud's symptom within the past 7 years before entry and a HRCT that showed fibrosis affecting at least 10% of the lungs. The primary end point was the annual rate of decline in FVC. Key secondary end points were absolute changes from baseline in the modified Rodnan skin score (MRSS) and in the total score on the St. George's Respiratory Questionnaire (SGRQ). Neither of the two secondary endpoints achieved statistical significance highlighting the variability and poor reproducibility of the MRSS and the questionable applicability of the SGRQ for understanding dyspnea in SSc-ILD. The adjusted annual rate of change in FVC was −52.4 ml per year in the nintedanib group and −93.3 ml per year in the placebo group (difference, 41.0 ml per year; 95% [CI], 2.9–79.0; *P* = 0.04). Patients on a stable MMF dose did not elicit further improvement with add-on therapy with nintenadib.

Diarrhea, the most common adverse event, was reported in 75.7% of the patients in the nintedanib group and in 31.6% of those in the placebo group (52). An extension trial, SENSCIS-ON will assess long-term safety of treatment with oral Nintedanib in 450 subjects who completed the SENSCIS trial. This trial should be completed by July 2021.

## Other Treatment Modalities

### Lung Transplantation

Lung transplantation should be considered in the early stage of respiratory failure for all patients with chronic lung disease. However, gastrointestinal comorbidities that are often seen in patients with SSc-ILD may complicate the transplant evaluation (80). A systematic review by Khan et al. was performed to identify studies of the survival outcome post lung transplantation between patients with SSc vs. patients with no Ssc (ILD patients requiring lung transplantation) (81). SSc post-transplantation survival ranged 69–91% at 30-days, 69–85% at 6-months, 59–93% at 1-year, 49–80% at 2-years, and 46–79% at 3-years (82–85). The short-term and intermediate-term survival post-lung transplantation are similar to ILD patients requiring lung transplantation.

### Autologous Hematopoietic Stem Cell Transplantation (AHSCT)

Autologous hematopoietic stem cell transplantation (AHSCT) has been proposed as a potential therapy for severe SSc disease (86). In a meta-analysis study including patients with SSc-ILD on cyclophosphamide who underwent AHSCT, AHSCT reduced all-cause mortality (risk ratio [RR], 0.5 [95% confidence interval, 0.33–0.75]) and improved FVC (mean difference [M] 9.58% [95% CI, 3.89–15.18]), total lung capacity (M, 6.36% [95% CI, 1.23–11.49]), and assessment of quality of life (QOL) using a Short Form Health Survey showed improvement (M, 6.99% [95% CI, 2.79–11.18]) (87). Treatment-related mortality considerably varied between trials, but was overall higher with AHSCT (RR, 9.00 [95% CI, 1.57–51.69]). In the ASSIST trial, HSCT and antithymocyte globulin therapy preceded by CYC and filgrastim was superior to CYC with regards to skin score and lung volumes, although no difference was observed in DLco No deaths occurred in either group over 24 months of follow up (88). Recently, the SCOT (Scleroderma: CYC or transplantation) trial in patients with severe dcSSc with renal or pulmonary involvement, which goal was to determine the safety and effectiveness of high dose immunosuppressive therapy followed by AHSCT compared to CYC alone. The study demonstrated that myeloablative CD34+ selected AHSCT promoted greater event-free survival (survival without significant organ damage or death) than 12 months of CYC. The survival benefit was also noted at 54 months (79 vs. 50%) and at 72 months (74 vs. 47%) (89). Tables 1, 2 show a summary of ongoing and completed clinical trials on Ssc-ILD treatment.

**Table 1:** Completed clinical trials for patients with SSc-ILD.

**Drug and study design**	**Name of study**	**Indications**	**Adverse effects**
**Mycophenolate mofetil (MMF)**. 2-year randomized, double-blind, active comparator/placebo-controlled trial	SLSII (57) NCT00883129	-First line treatment in patients who are at risk of progressive ILD. -Maintenance therapy	-Bone marrow suppression -Gastrointestinal (nausea, diarrhea, abdominal cramping) -Pancytopenia -Hypertension -Hyperglycemia
**Cyclophosphamide (CYC)** 1-year, randomized, double-blind, placebo-controlled trial plus 1 additional year of follow-up without study medication	SLS I (28, 59) NCT00004563	Second line treatment	-Infertility -Opportunistic infections -Hemorrhagic cystitis -Bladder cancer -Leukopenia -Thrombocytopenia
**Bosentan** 12-month randomized, double-blind, placebo-controlled trial	BUILD-2 (66) NCT00070590	Investigational approach	-Gastrointestinal (weight gain, nausea, vomiting) -Fatigue, Dizziness -Edema
**Pirfenidone** 16-week randomized, open-label comparison of two titration schedules	LOTUSS (76) NCT01933334	Investigational approach	-Gastrointestinal -Skin(sun sensitivity and rash) - Elevated liver enzymes
**Pomalidomide** 52 week randomized, double-blind, placebo-controlled, parallel-group study	CC-4047 (1, 74) NCT01559129	Investigational approach	-Gastrointestinal -Leuokopenia
**Nintedanib** 52 week, double blind, randomized, placebo-controlled trial evaluating FVC changes, efficacy and safety	SENCSIS trial (52, 79) NCT02597933	Investigational approach	-Gastrointestinal, mainly diarrhea -High blood pressure
**Hematopoietic bone marrow stem cell transplant** Randomized, open-label, phase II multicenter study of high-dose immunosuppressive Therapy	Scleroderma: cyclophosphamide or transplantation (SCOT) NCT00114530	Investigational approach	-Immunosuppression

**Table 2:** Ongoing clinical trials for SSc-ILD patients.

**Drug and study design**	**Name of study**	**Clinical trial identifier**	**Phase trial**
**Nintedanib** An open-label extension trial of the long term safety of Nintedanib.	SENCSIS trial	NCT03313180	III
**Bortezomib**	Comparing and combining Bortezomib and Mycophenolate in SSc pulmonary fibrosis	NCT02370693	II, recruiting
**Pirfenidone plus MMF vs. MMF plus placebo**.	Scleroderma Lung Study III	NCT03221257	II, recruiting

## Conclusions

Although there is no consensus statement that defines the criteria for SSc-ILD, HRCT, and PFTs serve as the primary diagnostic and staging parameters for establishing a diagnosis. Although MMF has been the initial treatment choice for SSc-ILD due to safer toxicity profiles and outcomes, more recent trials raise the option of antifibrotics or combination immunomodulatory/antifibrotic therapy as potential new treatments for patients with SSc-ILD. Lung transplant should be considered as an option, but the significant comorbidities associated with SSc including GI comorbidities should be addressed with medical and surgical evaluations prior to referring for transplant.

Many questions remain unanswered. When should treatment be initiated for SSc-ILD? What treatment regimen is most efficacious? How long should the patient be treated with SSc-ILD? With the development of more sophisticated classification criteria and assessment of HRCT, availability of reliable and reproducible biomarkers and molecular profiling, answers for these questions will impact treatment strategies for patients with SSc-ILD.

## Author Contributions

PB conducted literature review, conducted exploratory analysis, and helped to develop the first draft of the manuscript. MG helped in developing the first draft of the manuscript. MM conducted literature review, conducted exploratory analysis, and developed the final version of the manuscript.

### Conflict of Interest

The authors declare that the research was conducted in the absence of any commercial or financial relationships that could be construed as a potential conflict of interest.
